# Cross-Talk between Fibroblast Growth Factor Receptors and Other Cell Surface Proteins

**DOI:** 10.3390/cells8050455

**Published:** 2019-05-14

**Authors:** Marta Latko, Aleksandra Czyrek, Natalia Porębska, Marika Kucińska, Jacek Otlewski, Małgorzata Zakrzewska, Łukasz Opaliński

**Affiliations:** Department of Protein Engineering, Faculty of Biotechnology, University of Wroclaw, Joliot-Curie 14a, 50-383 Wroclaw, Poland; marta.latko2@uwr.edu.pl (M.L.); aleksandra.czyrek@uwr.edu.pl (A.C.); natalia.porebska2@uwr.edu.pl (N.P.); kucinska.marika@gmail.com (M.K.); jacek.otlewski@uwr.edu.pl (J.O.); malgorzata.zakrzewska@uwr.edu.pl (M.Z.)

**Keywords:** fibroblast growth factor receptors, signaling, receptor cross-talk, coreceptor, membrane proteins

## Abstract

Fibroblast growth factors (FGFs) and their receptors (FGFRs) constitute signaling circuits that transmit signals across the plasma membrane, regulating pivotal cellular processes like differentiation, migration, proliferation, and apoptosis. The malfunction of FGFs/FGFRs signaling axis is observed in numerous developmental and metabolic disorders, and in various tumors. The large diversity of FGFs/FGFRs functions is attributed to a great complexity in the regulation of FGFs/FGFRs-dependent signaling cascades. The function of FGFRs is modulated at several levels, including gene expression, alternative splicing, posttranslational modifications, and protein trafficking. One of the emerging ways to adjust FGFRs activity is through formation of complexes with other integral proteins of the cell membrane. These proteins may act as coreceptors, modulating binding of FGFs to FGFRs and defining specificity of elicited cellular response. FGFRs may interact with other cell surface receptors, like G-protein-coupled receptors (GPCRs) or receptor tyrosine kinases (RTKs). The cross-talk between various receptors modulates the strength and specificity of intracellular signaling and cell fate. At the cell surface FGFRs can assemble into large complexes involving various cell adhesion molecules (CAMs). The interplay between FGFRs and CAMs affects cell–cell interaction and motility and is especially important for development of the central nervous system. This review summarizes current stage of knowledge about the regulation of FGFRs by the plasma membrane-embedded partner proteins and highlights the importance of FGFRs-containing membrane complexes in pathological conditions, including cancer.

## 1. Introduction

**Figure 1:**
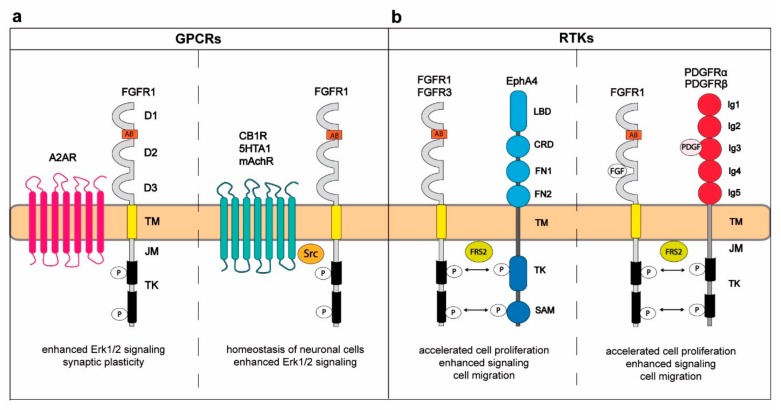
(**a**) Interplay between fibroblast growth factor receptors (FGFRs) and G-protein-coupled receptors (GPCRs) (**a**) and other receptor tyrosine kinases (RTKs) (**b**) in the regulation of downstream signaling. The extracellular region of FGFRs is composed of immunoglobulin like domains D1–D3 (gray) and the acidic box (AB; red). FGFRs are anchored in the plasma membrane by a single transmembrane helix (yellow). The cytosolic part of FGFRs consists of the juxtamembrane domain (JM) and the split tyrosine kinase domain (TK; black). GPCR–FGFR complexes may involve Src as a mediator between receptors or form functional heterocomplexes without involvement of Src. (**b**) FGFRs interact with other RTK members in the plasma membrane and can be directly activated by intracellular tyrosine kinase domains of partner proteins like Eph receptors or PDGFRs. EphA4 receptor contains the N-terminal ligand binding domain (LBD) followed by the cysteine rich domain (CDR) and two fibronectin type III domains (FN1–2). EphA4 is embedded in the membrane by a single transmembrane domain (TM). The cytosol-oriented region of EphA4 is composed of the tyrosine kinase domain (TK) and the sterile alpha motif (SAM). The TK domain of EphA4 interacts with JM region of FGFRs. PDGFRs contain five immunoglobulin-like domains (Ig1–Ig5) in their extracellular region, a single transmembrane span (TM), and intracellular juxtamembrane (JM) and tyrosine kinase (TK) domains. TK of PDGFRs directly phosphorylates FGFRs.

Fibroblast growth factor receptors 1–4 (FGFR1–4) form a group of receptor tyrosine kinases (RTKs) that are present on the surface of various cell types. FGFRs govern plethora of key cellular processes, including proliferation, migration, differentiation, and apoptosis, and their proper functioning is critical for development of the human body and homeostasis [1]. Alterations in FGFR1–4 are frequently detected in variety of developmental diseases and cancers, like prostate, breast, lung, and ovarian cancers [2,3]. The overall structure of FGFRs is typical for RTKs with an N-terminal region including three immunoglobulin-like domains D1–D3 exposed to the extracellular space, a single transmembrane span and a cytosolic tyrosine kinase domain (Figure 1a) [1,4]. The extracellular part of FGFRs constitutes binding sites for their natural ligands, FGFs, heparan cofactors, and a number of partner proteins [5,6]. Additionally, the ectodomain of FGFRs includes several motifs that prevent receptor autoactivation in the absence of growth factors [7,8,9,10]. The transmembrane helix of FGFRs anchors the receptors in the membrane and facilitates dimerization [11]. In the cytosol, the juxtamembrane (JM) region of FGFRs is involved in receptor dimerization and moderates transmission of signals [12,13,14]. The initiation of intracellular signaling circuits requires activation of FGFRs split kinase domain [1,5]. FGFR1–3 are subjected to alternative splicing in their extracellular region, yielding b and c isoforms of the receptors that differ in expression pattern and ligand specificity [15,16,17]. The FGFR family includes also fifth member—FGFRL1 (FGFR5)—which is homologous to FGFRs in the extracellular region, but lacks the cytosolic tyrosine kinase domain [18,19].

Classically, the transmission of signals through the plasma membrane via FGFRs requires binding of appropriate growth factors and subsequent receptor activation. The canonical FGFs (FGF1–FGF10, FGF16, FGF17, FGF18, FGF20, and FGF22) are effective ligands in FGFRs binding and activation. In an inactive state monomeric FGFRs bind canonical FGFs, which triggers conformational changes in the receptor, resulting in dimerization and transactivation of cytosolic tyrosine kinases [1,20]. Sequential phosphorylation of tyrosine residues within the cytosolic tail of FGFRs creates docking sites for downstream signaling proteins [1,21]. The signals are further propagated through several pathways: Ras/Raf-mitogen-activated protein kinase/extracellular signal regulated kinase kinase (MEK)–extracellular signal regulated kinase (ERK), phosphoinositide 3-kinase (PI3K)/protein kinase B (AKT)/mammalian target of rapamycin (mTOR), phospholipase Cγ (PLCγ), and signal transducer and activator of transcription (STAT) [1,20]. 

FGFR-dependent signaling can be adjusted in several ways, including the diversified tissue distribution, different expression level of signaling components and their alternative splicing, which influences tissue development and disease progression [1]. Transmission of signals can be further modulated by ligand type, as FGFR complexes with different FGFs may vary in the strength and duration of propagated signals, which in turn decides cell fate [20,22]. FGFRs signaling can be modified as well by spontaneous receptor dimerization in the absence of ligands [23]. The posttranslational modifications, like glycosylation, ubiquitination, and phosphorylation, influence ligand binding and constitute negative feedback mechanisms for inhibition of FGFRs signaling [24,25,26,27,28]. Additionally, the cellular trafficking of FGFRs may regulate signals specificity, intensity, and timing [29,30,31]. 

One of the emerging means to modulate FGFRs activity is via formation of complexes with other plasma membrane proteins. Assembly of such complexes can be critical for transmission of signals, which is the case for endocrine FGFs (FGF19, FGF21, and FGF23) [32]. Partner proteins may deliver cofactors that facilitate formation of productive signaling modules or regulate the cellular transport of FGFRs [1]. Distinct types of cell surface receptors interact with FGFRs, leading to integration of different signaling routes or modulation of signal transmission. Several high throughput studies led to the discovery of numerous potential interaction partners of FGFRs within the plasma membrane [33,34,35]. However, the biological significance for most of them still needs to be elucidated. 

In the next chapters we focus on the interplay between FGFRs and their binding partners in the regulation of signaling and cell behavior.

## 2. Cross-Talk between FGFRs and G-Protein-Coupled Receptors in Regulation of the Central Nervous System 

G-protein-coupled receptors (GPCRs) constitute one of the largest groups of receptors responsible for signal transmission [36,37,38]. GPCRs are composed of an N-terminal extracellular domain, seven transmembrane helices, and a C-terminal region directed to the cytosol. Stimulation of GPCRs by extracellular ligands induces conformational changes within GPCRs, triggering intracellular signaling pathways modulated by heterotrimeric G proteins [39,40]. Due to their wide diversity GPCRs modulate numerous processes, including, among others, nervous system transmission, visual, gustatory and smell sensing, inflammation, and recognition of cell density [41]. 

Various members of GPCRs and RTKs form heterocomplexes, which trigger intracellular signaling and cellular response different from that induced by RTKs or GPCRs alone [42]. The alterations in transmitted signals by GPCRs-RTKs heterocomplexes is achieved by the transactivation of RTKs by GPCRs which may occur via two distinct mechanisms: one relying on GPCRs activation and signaling that results in release of RTKs ligands and subsequent RTKs activation and second mechanism that involves a direct interaction and subsequent activation of RTKs by GPCRs [42]. The transactivation of RTKs by GPCRs was already demonstrated for a large number of RTKs, including epidermal growth factor receptors (EGFRs), platelet-derived growth factor receptors (PDGFRs), and insulin-like growth factor receptors (IGFRs) [42]. 

In the central nervous system (CNS), GPCR-dependent signaling controls proliferation, migration, survival, and differentiation of neurons [43]. FGFRs are expressed in different areas of brain. While FGFR1 is widely found in the hippocampus and in various parts of the cortex, FGFR2 and FGFR3 proteins are scattered throughout the CNS, and their expression profile changes with the brain development. FGFR4 is less abundant than other FGFRs and is mainly localized to the medial habenular nucleus [44,45,46,47,48]. The FGFRs are involved in the development, function and maintenance of the CNS [49]. Yeast two-hybrid (Y2H) screens revealed FGFR1 as a binding partner of G-protein-coupled receptor (GPCR)–adenosine receptor A2AR. The FGFR1-A2AR interaction was further confirmed by pull-down and coimmunoprecipitation [50]. The simultaneous stimulation of PC12 cells with A2AR agonist and FGF2 results in enhanced activation of downstream signaling pathways in comparison to single treatments, pointing on the synergistic effect of both receptors on cellular signaling. The enhanced activation of extracellular regulated kinases 1/2 (ERK1/2) requires assembly of the FGFR1-A2AR complex, pointing on the functional relevance of this interaction. The modulation of signaling by FGFR1-A2AR heterocomplexes was found to be important for regulation of the synaptic plasticity (Figure 1a) [50]. 

Cannabinoid receptor 1 (CB1R) is GPCR-ubiquitous in neurons, mediates the biological action of endogenous and synthetic cannabinoids, and regulates homeostasis of neuronal cells [51]. CB1R-FGFR1 interaction in neurons was demonstrated by means of coimmunoprecipitation. CB1R induces the transactivation of FGFR1 via protein kinase C (PKC) that in turn activates Fyn and Src. The latter proteins trigger activation of FGFR1 by phosphorylating key tyrosine residues of the receptor kinase domain [52]. The formation of CB1R-FGFR1 complexes occurs in lipid rafts of the plasma membrane, leads to activation of ERK1/2, and is important for neuronal differentiation (Figure 1a). 

Using the proximity ligation assay (PLA) the interaction of FGFR1 with muscarinic acetylocholine receptor (mAChR) subtype M1R was visualized [53]. Upon stimulation of hippocampal neurons with M1R agonist oxotremorine-M the activation of FGFR1 was observed. The exact mechanism of FGFR1 transactivation is not clear, however it involves Src tyrosine kinase that phosphorylates FGFR1 [53]. The cross-talk between mAChR and FGFR1 enhances neurite growth (Figure 1a) [53].

Binding between FGFR1 and 5-hydroxytriptamine receptor 1A (5-HT1A) was also demonstrated with PLA, but it was further confirmed by coimmunoprecipitation and bioluminescence resonance energy transfer (BRET) in a wide variety of cell types [54,55,56]. The number of FGFR1-5-HT1A complexes increases upon stimulation of cells with the FGF2 and 5-HT1A agonist 7-(Dipropylamino)-5,6,7,8-tetrahydronaphthalen-1-ol (8-OH-DPAT), confirming the functional interplay between these receptors [55]. Activation of 5-HTA1 with 8-OH-DPAT causes subsequent FGFR1 phosphorylation mediated by Src [55]. The simultaneous activation of FGFR1 and 5-HTA1 results in synergistically enhanced signaling that induces growth and controls homeostasis of neuronal cells (Figure 1a) [55]. Interestingly, the FGFR1–5-HT1A heterocomplexes display anti-depressive effects and thus may constitute targets for treatment of mood disorders [55,57,58,59]. 

Mu-opoid receptor (MOR) binds with high affinity to enkephalins and endorphins that modulate neuronal excitability. In rat glioma C6 cells MOR induces rapid activation of ERK1/2 via the transactivation of FGFR1. Again, the exact mechanism of this transactivation is unknown. Also the direct interaction between MOR and FGFR1 has not been yet demonstrated [60].

Summarizing, various members of GPCRs affect activity of FGFRs through the transactivation, which usually requires formation of the direct interaction between these receptors and involves Src as a bridging factor. The cross-talk between GPCRs and FGFRs is especially relevant for the development and functioning of neurons. GPCRs constitute large group of receptors, however only few members of the GPCRs family were demonstrated to bind FGFR1. The function of one type of receptors can be modulated by binding to other group of receptors. Since GPCRs play diverse pivotal functions in cells, the involvement of FGFRs in the regulation of GPCRs needs to be elucidated.

## 3. Interplay between FGFRs and Other RTKs 

Diversification of signals transmitted by FGFRs can be also achieved by the interplay with other members of RTK family. The cross-talk between RTKs can occur via formation of receptor heterocomplexes and subsequent tyrosine phosphorylation of one receptor by tyrosine kinase of the other one. Alternatively, the transphosphorylation of RTKs in the complex can be mediated by the cytosolic kinase, like Src [61]. 

Eph receptors are activated by ephrin ligands and constitute the largest family of RTKs [62,63]. Based on sequence similarity and preference for ephrins A or B, Eph receptors are divided into EphA (EphA1–EphA10) and EphB (EphB1–EphB6) receptors [64]. The Eph receptors contain structural features characteristic for RTKs: an extracellular ligand binding region, a transmembrane domain, and an intracellular tyrosine kinase module [65]. The N-terminal extracellular part of Eph receptors is composed of ephrin binding domain followed by the cysteine rich EGF-like motif and two fibronectin type III repeats (FN3) FN1 and FN2. The cytosolic region of Eph receptors includes the juxtamembrane domain, the tyrosine kinase and the sterile alpha motif (SAM) (Figure 1b) [66]. Remarkably, activation of Eph receptors by ephrins requires the assembly of cell to cell contacts, as ephrins are embedded in the plasma membrane by the glycosylphosphatidylinositol (GPI) anchor (ephrins A) or the transmembrane helix (ephrins B) [64]. Binding of Eph receptor to ephrin present on the surface of aligned cell is followed by the juxtaposition of cytoplasmic kinase domain that evokes the transphosphorylation of receptor tyrosine residues initiating downstream signaling cascades [67]. The Eph receptor–ephrin complexes can be further arranged into high order assemblies that modulate cellular signaling [68,69]. The Eph receptor–ephrin complexes adjust cell adhesion, organization of cytoskeleton, angiogenesis, neural development, and plasticity [70].

EphA4 receptor emerged as binding partner of FGFR3 in Y2H screens [71]. Further experiments, including coimmunoprecipitation revealed that the tyrosine kinase domain of Eph4 directly interacts with the JM domain of FGFR1–4 [71]. The formation of EphA4-FGFR complexes requires phosphorylation of tyrosine residues within JM domain of Eph4. Kinase domains of EphA4 and FGFRs can transphosphorylate each other. Furthermore, EphA4 ligand ephrin-A1 enhances FGFRs signaling, indicating significance of the FGFRs transactivation by EphA4 for the modulation of intracellular signal propagation [72]. Signals transmitted via FGF2/FGFR1/EphA4 complexes are enhanced in relation to FGF2/FGFR1, resulting in accelerated cell proliferation and migration [67]. In addition, the interaction between EphA4 and the fibroblast growth factor receptor substrate 2 alpha (FRS2α), a protein required for FGFRs signaling [73] was demonstrated with Y2H and pull down experiments. Noteworthy, the ternary complex, involving FGFR1, EphA4, and FRS2α was detected. Thus, FRS2α acts as a tethering molecule that integrates signals from both receptors and regulates self-renewal, differentiation, and proliferation of neural stem/progenitor cells [74,75]. The cross-talk between Eph and FGFRs and Eph receptors was further confirmed by the observation that FGFRs phosphorylate EphA receptor target molecule, ephexin-1 [76]. Furthermore, Dlg-1, a scaffolding protein directly interacting with EphA receptors, can modulate FGFRs signaling (Figure 1b) [77,78]. 

Platelet-derived growth factor receptors alpha and beta (PDGFRα and PDGFRα) are RTKs that are activated by five different platelet-derived growth factors (PDGF): PDGF-AA, PDGF-BB, PDGF-AB, PDGF-CC, and PDGF-DD [79,80]. Through regulation of cellular signaling PDGFRs influence cell motility, proliferation, and angiogenesis and aberrant PDGFRs are implicated in cancer [79]. PDGFRs are composed of the extracellular region divided into five Ig-like domains, from which Ig2 and Ig3 form the PDGF binding site, a single transmembrane span, and the intracellular tyrosine kinase domain (Figure 1b) [81,82]. In vitro and in vivo experiments using solid-phase assay (SPA), coimmunoprecipitation, and Förster Resonance Energy Transfer (FRET) revealed that PDGFRα interacts with high affinity with FGFR1 [83]. The formation of PDGFRα-FGFR1 complexes is facilitated by the presence of ligands for both receptors [83]. The interaction between PDGFRβ and FGFR1 was demonstrated by means of coimmunoprecipitation [84]. In this receptor heterocomplex PDGFRβ directly phosphorylates FGFR1 on tyrosine residues [84]. Interestingly, FRS2 functions as a bridging molecule between PDGFRβ and FGFR1 (Figure 1b) [84]. The interplay is not only observed between the receptors but also at the level of their ligands. PDGF-BB and FGF2 interact with each other and activity of individual ligands in PDGF-BB-FGF2 complex is altered [85,86,87]. Remarkably, PDGFRs and FGFRs are often dysregulated in cancer and are targets of numerous therapeutic approaches [88].

Summarizing, FGFRs assemble into large multiprotein complexes with other RTK members and accessory proteins. The tyrosine kinase domains of different RTKs are able to transphosphorylate each other, initiating signals and adjusting their strength and specificity. Importantly, the interplay between RTKs is often coordinated at the level of FRS2. The fact that different members of RTKs can transactivate each other suggests the presence of an additional level of complexity in RTKs signaling. The family of RTKs is composed of 58 members; however, to date only few RTKs have been implicated in the FGFRs transactivation. Further studies on the interplay of FGFRs with other RTKs may uncover novel cellular regulatory mechanisms. Numerous FGFR-targeted anticancer therapies aim on the inhibition of FGFs interaction with FGFRs. Since FGFRs can be activated by other receptors in the absence of ligands, the detailed knowledge about FGFRs interplay with other RTKs may help in the development of novel therapeutics downregulating FGFRs signaling. 

## 4. Modulation of FGFRs Activity by Cell-Surface Proteins Involved in Adhesion

**Figure 2:**
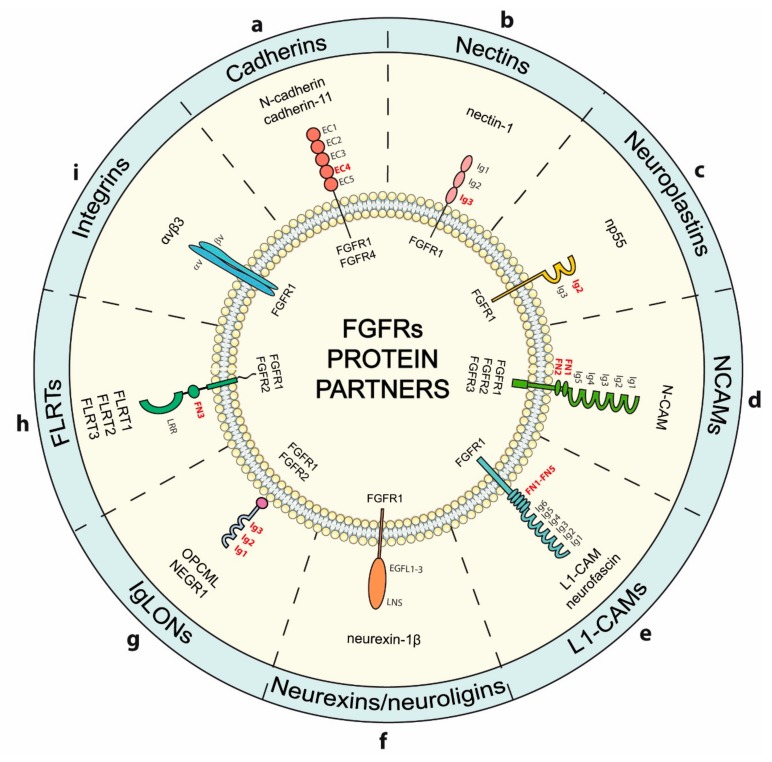
Cross-talk between FGFRs and various cell adhesion molecules. The interaction of particular FGFR with members of CAMs subgroup is indicated. The domain architecture of FGFR partner proteins is shown. Domains (where identified) responsible for the interaction between the partner protein and FGFR are indicated in red. (**a**) Cadherins reported to interact with FGFR1 and FGFR4 contain five EC domains in their extracellular region, a single transmembrane helix, and a cytosoilc tail interacting with several signaling proteins. (**b**) Nectins are composed of three immunoglobulin-like domains Ig1–Ig3, a single transmembrane domain, and a cytosolic region. Nectins bind FGFR1 using the Ig3 domain (**c**) Neuroplastin (Np55) contains two immunoglobulin-like Ig1–Ig2 domains in their extracellular region and are embedded in the membrane by a single transmembrane helix, exposing short tail into the cytosol. Np55-FGFR1 interaction involves the Ig2 domain of Np55 (**d**) NCAMs expose on the surface of the cells five immunoglobulin-like domains Ig1–Ig5 and two fibronectin type III domains FN1 and FN2. The cytosolic tail of NCAMs varies in length. NCAMs bind FGFR1-FGFR3 using FN1–FN2 domains (**e**) L1-CAM is a single spanning plasma membrane protein with six Ig-like domains (Ig1–Ig6) and five fibronectin type III domains (FN1–FN5) in its extracellular region. FGFR1 binding requires the FN1–FN5 region of L1-CAM (**f**) Neurexins contain different numbers of the laminin-neurexin-sex hormone binding globulin domains (LNS) and three EGF-like domains (EGFL1–3), a single transmembrane span and the cytosolic tail interacting with cytoskeletal and signaling proteins. The extracellular region of neurexin 1-β interacts with FGFR1. (**g**) Ig-LON family members: OPCML and NEGR1 interact with FGFR1 and FGFR2. Ig-LON proteins contain three immunoglobulin-like domains Ig1–Ig3 that are implicated in FGFR binding. (**h**) FLRTs are single spanning transmembrane proteins containing the leucine-rich repeat domain (LRR) and the FN3 domain in their extracellular region. FLRTs employ the FN3 domain for FGFR1 and FGFR2 binding. (**i**) Integrins are composed of different α and β subunits. Integrin αvβ3 forms complexes with FGFR1.

Establishing cell-cell contacts requires an extensive remodeling of cellular components. Communication between cells involves interactions that are mediated by various cell adhesion molecules (CAMs). At the cell-cell interface extensive signaling is triggered, which coordinates remodeling of cellular structures. Noteworthy, FGFRs emerged as CAMs binding partners that participate in the signaling initiated by CAMs at cell-cell contacts (Figure 2).

### 4.1. Cadherins

Cadherins are integral membrane proteins that are involved in the formation of specific cell-cell contacts, the adherens junctions (AJs) [89]. AJs are regulated by the alternative splicing of cadherins and are important for tissue development, homeostasis of epithelium and are implicated in different types of cancer [90,91,92]. Cadherins on opposing cells interact with each other via extracellular regions composed of five domains (EC1–EC5) in a calcium-dependent manner (Figure 2a) [92]. The cytosolic tail of cadherins binds catenins and other intracellular factors that link cadherin complexes to the cytoskeleton and forms signaling platforms at the cell-cell interface [90]. 

Neuronal cadherin (N-cadherin, cadherin-2) is expressed in various cell types, but its highest level is detected in neuronal and mesenchymal cells, where it coordinates cell migration and proliferation [93]. The functional interaction between N-cadherin and FGFRs was demonstrated in numerous cells, where N-cadherin was shown to activate FGFRs and receptor-downstream signaling (Figure 2a) [94,95,96]. The interaction between N-cadherin and FGFR1 was demonstrated by means of coimmunoprecipitation in different cell lines [97,98]. The binding studies with truncated variants of FGFR1 revealed that the acidic box of the receptor extracellular region is required for the interaction with N-cadherin [97,98]. Fluorescence microscopy analyses revealed that in transfected NIH3T3 cells N-cadherin and FGFR1 colocalize at the plasma membrane, however the N-cadherin-FGFR1 complexes are less abundant at the cell-cell contact sites where N-cadherin is enriched, suggesting dynamic nature of this interaction [97]. Formation of N-cadherin complexes with FGFR1 in breast cancer cells causes decreased internalization and lysosomal degradation of FGFR1, and sustained receptor signaling via MAPKs. Thus, N-cadherin may promote invasiveness of cancer cells not only by regulating cell-cell interactions, but also by affecting FGFR1 levels and activity [98,99,100]. Silencing of N-cadherin results in the accelerated FGFR1 degradation, whereas overproduction of N-cadherin is accompanied by increased levels of FGFR1. Thus, N-cadherin stabilizes FGFR1 and simultaneously enhances FGF2-induced proliferation and differentiation of epiblast stem cells [101]. Using coimmunoprecipitation, the interaction of N-cadherin with FGFR4 was demonstrated in pancreatic tumor cells and was dependent on neural cell adhesion molecule (N-CAM) [102]. Moreover, FGFR4-388Arg mutant frequently observed in various cancers induces signaling cascades that lead to enhanced N-cadherin expression and modulates epithelial to mesenchymal transition (EMT) [103].

Cadherin-11 is widely expressed in mesenchymal cells like osteoblasts and neurons, and is important for tissue development during embryogenesis [104,105]. It is implicated in migration of cancer cells and in epithelial to mesenchymal transition [106,107,108,109]. The formation of complexes between FGFR1 and the cadherin-11/β-catenin adhesion complexes was demonstrated by coimmunoprecipitation (Figure 2a) [110]. Pull-down experiments revealed that the cadherin-11-FGFR1 interaction occurs through their extracellular domains. Cadherin-11 initiates intracellular signaling pathways via FGFR1 and recruits FGFR1 into areas of cell-cell contacts [49]. The cadherin-11-induced FGFR1 signaling stimulates neurite outgrowth [49]. 

### 4.2. Nectins

Nectins comprise a group of four plasma membrane proteins (Nectin-1–4) involved in formation of cell-cell contacts that are relevant in the neural development and disorders, and cancer [111]. Nectins contain an extracellular region composed of three immunolglobulin-like (Ig) domains, a single transmembrane helix, and a cytosolic domain (Figure 2b). Nectins from one cell can oligomerize in trans orientation with nectins present on the opposing cell, which results in cell adhesion. Depending on the involvement of accessory proteins nectins can be involved in establishing several types of adhesion complexes [111,112]. Using surface plasmon resonance (SPR) a direct interaction between Ig2–Ig3 domains of FGFRs and Ig3 of nectin-1 was demonstrated (Figure 2b). Binding of Ig3 of nectin-1 to FGFR1 results in receptor activation. Nectin-1 induces neurite outgrowth in hippocampal neurons in FGFR1-dependent manner, indicating that nectin-1 co-clusters with FGFR1 at the cell–cell contacts to stimulate differentiation and development of neurons [113]. 

### 4.3. Neuroplastins

Neuroplastins are cell adhesion molecules from immunoglobulin superfamily [114]. Neuroplastin Np55 is expressed in numerous cell types and tissues [115]. Np55 contains two Ig-like domains—Ig2 and Ig3—oriented towards the extracellular space, a single transmembrane span, and a short cytoplasmic tail (Figure 2c) [116]. SPR analysis revealed that Np55 directly interacts with the Ig2–Ig3 region of FGFR1 (Figure 2c). Binding of Np55 to FGFR1 present on the cell surface leads to receptor activation and initiation of downstream signaling. Although FGF2 and Np55 bind to the same region of FGFR1, these proteins elicit different effects on the receptor. Np55-FGFR1 complexes stimulate neurite outgrowth in primary hippocampal neurons, while FGF2-FGFR1 does not, which suggests different mode of intracellular signaling activation by these two FGFR1 ligands. Peptide based on Np55 extracellular domain was able to activate FGFR1 and downstream signaling and displayed antidepressant effects [117]. 

### 4.4. N-CAMs

Neural cell adhesion molecules (N-CAMs) are cell surface glycoproteins involved in axonal growth, cell migration, synaptic plasticity, and cell differentiation, and are implicated in various diseases including cancer [118,119]. N-CAMs contain five Ig-like domains and two FN3 domains in their extracellular region. NCAM-140 and NCAM-180 are embedded in the plasma membrane via transmembrane helices and display cytoplasmic tails of different length (Figure 2d). In contrast NCAM-120 utilizes the glycosylphosphatidylinositol (GPI) moiety for attachment to the cell surface [120]. 

The functional interplay between FGFRs and N-CAMs in neurite outgrowth was initially demonstrated by Williams et al. [94]. Subsequent studies confirmed a direct interaction of N-CAMs and FGFRs in different types of cells, including cancer cells [97,102,121,122,123]. The FN3 domains are responsible for the N-CAMs interaction with the Ig2–Ig3 region of FGFRs (Figure 2d) [124,125,126]. N-CAMs bind to FGFR1-FGFR3, but not to FGFR4, and these interactions depend on the receptor splice variants [127]. Binding of N-CAMs to FGFRs results in activation of the receptor and initiation of signaling cascades. The N-CAMs-FGFRs interplay is important for neuronal tissue development, but is also implicated in cancer. The N-CAMs/FGFRs complexes are observed in epithelial ovarian carcinoma, where they stimulate cancer cell migration and invasion [128,129]. The N-CAMs/FGFRs signaling may also modulate EMT [130]. Interestingly, N-CAMs can affect the cellular trafficking of FGFRs. Activation of FGFR1 by FGFs triggers receptor internalization and lysosomal degradation. In contrast, N-CAM-FGFR1 complexes are internalized, but the majority of the receptor is recycled from endosomes to the cell surface [121]. This differential FGFR1 cellular transport determines distinct cell fate depending on stimulation with FGF or N-CAM proteins [73]. 

### 4.5. L1-CAMs

L1-CAM is a cell surface glycoprotein that contains six Ig-like domains and five FN3 motifs in its extracellular region, a single TM span, and an intracellular tail that binds several signaling proteins (Figure 2e) [118]. The functional link between FGFR1 and L1-CAM was established by the observation that extracellular region of L1-CAM activates FGFR1, stimulating neurite outgrowth [94]. SPR experiments demonstrated a direct interaction between L1-CAM FN3 domains 1–5 and FGFR1 Ig2–Ig3 domains that was dependent on ATP [131]. Noteworthy, the cross-talk between FGFR1 and L1-CAM plays a role in proliferation and motility of glioma cells. The soluble, extracellular region of L1-CAM is often released by the cells due to the limited proteolysis involving ADAM-10 protease [132]. By binding to FGFR1 the extracellular region of L1-CAM leads to receptor activation, resulting in stimulation of glioma cell proliferation and motility [133]. The multiprotein complex of L1-CAM, FGFR1, and secreted glycoprotein Anosmin-1, which is involved in cell adhesion, motility, and differentiation, were also implicated in neurite branching [134,135,136,137,138,139].

Neurofascins are L1-CAM group members that control neurite outgrowth and synaptic organization [140]. The interaction between neurofascin (isoform NF166) and FGFR1 was demonstrated by coimmunoprecipitation [141]. Experiments with truncated versions of neurofascin revealed presence of two binding sites for FGFR1: an extracellular and an intracellular. Nevertheless, only the intracellular region of neurofascin is critical for FGFR1-dependent neurite outgrowth [141,142]. 

### 4.6. Neurexins

Neurexins and neuroligins are neuronal CAMs that regulate synaptic organization and function [143,144]. Presynaptic neurexins consist of the extracellular region containing from one to six laminin- neurexin-sex hormone binding globulin domains (LNS) and three epidermal growth factor like (EGF-like) domains, O-glycosylation sites, a single transmembrane span, and the cytosolic region recruiting various intracellular cytoskeletal and signaling proteins (Figure 2f) [144]. Postsynaptic neuroligins are composed of the extracellular acetylcholinesterase-like domain, a region enriched in glycosylation sites, a single transmembrane helix and the C-terminal intracellular PDZ domain recognition motif. Neurexins and neuroligins form trans-synaptic tethers that organize structure and function of synapses [144]. SPR experiments revealed a direct interaction between extracellular domain of FGFR1 and ectodomain of neurexin-1β (Figure 2f) [145]. Neurexin-1β binding leads to the activation of FGFR1 and receptor-downstream signaling cascades in a dose-dependent manner [145]. 

### 4.7. IgLONs

IgLONs are CAMs from immunoglobulin superfamily composed of three Ig-like domains that are attached to the cell membrane via GPI anchor (Figure 2g) [146]. Neuronal growth regulator 1 (NEGR1) is IgLON member that regulates neuronal maturation [147]. The functional interplay between NEGR1 and FGFRs in neuronal development and disease was initially suggested by Pischedda et al. and Casey et al. [148,149]. This was further confirmed by detection of the interaction between extracellular regions of NEGR1 and FGFR2 (Figure 2g). NEGR1 influences FGFR2 intracellular trafficking, favoring receptor recycling. The prolonged intracellular trafficking of FGFR2 in endosome compartments results in enhanced receptor-dependent signaling. Importantly, it was demonstrated that the coordinated cortical development requires the functional interplay between FGFR2 and NEGR1 [150]. 

Opioid binding protein cell adhesion molecule (OPCML) is another IgLON member linked with FGFRs. OPCML is a tumor suppressor implicated in various cancers [151,152,153,154,155]. Coimmunoprecipitation revealed that OPCML interacts with FGFR1. Furthermore, pull down experiments with recombinant OPCML and FGFR1 truncations showed that the Ig1–Ig3 region of OPCML directly interacts with the extracellular domain of FGFR1 (Figure 2g). Binding of OPCML to FGFR1 and a few other RTK members results in their downregulation, which is likely a result of their altered intracellular trafficking and decreased recycling [156].

### 4.8. FLRTs

Fibronectin leucine-rich transmembrane (FLRTs) proteins comprise a group of three cell surface glycoproteins involved in cell adhesion during vascularization and synapse development [157,158,159,160,161]. FLRTs contain the N-terminal extracellular region composed of the leucine-rich repeat domain (LRR) and the FN3 domain. FLTRs are embedded in the cell membrane via a single transmembrane helix and contain a short cytoplasmic tail (Figure 2h) [162]. FLRTs mediate cell-cell contacts mainly through the interaction of LRR domains of FLRTs on neighboring cells or with latrophilin [157,162]. Coimmunoprecipitation, pull-down and BRET experiments revealed that the FN3 domain of FLRT2 and FLRT3 interacts with FGFR2 and FGFR1, respectively (Figure 2h) [162,163]. Assembly of the FLRT-FGFR complexes is mediated by the interaction between intracellular regions of these proteins [164,165]. FGFR1-dependent signaling leads to the tyrosine phosphorylation of the intracellular tail of FLRT1. In addition, formation of the FLRT1-FGFR1 complexes enhances receptor signaling upon stimulation with FGF ligand, which accelerates neurite outgrowth in MAPK-dependent manner [166].

### 4.9. Integrins

Integrins are adhesion molecules that recognize ligands present in the extracellular matrix and on the cell surface, playing a key role in establishing cell contacts and regulating intracellular signaling [167]. Subunits α (18 isoforms) and β (8 isoforms) assemble into 24 functional integrins that vary in terms of ligand specificity and cellular function (Figure 2i) [168]. Integrin-dependent signaling modulates survival, migration, and differentiation of cells [169]. Dysregulation of integrin adhesion complexes is widely implicated in various cancer types [170]. Coimmunoprecipitation experiments confirmed assembly of the ternary complex containing FGF1, FGFR1 and integrin αvβ3, with FGF1 acting as a bridging factor (Figure 2i). These multiprotein complexes are important for sustained activation of FGFR1-dependent kinases ERK1/2 [171]. Interestingly, the integrin binding-deficient mutant of FGF-1 (R50E) is capable of binding and activating FGFR1, however it fails to induce cell proliferation and migration, pointing on the functional relevance of integrin αvβ3 in FGF1 action [172,173]. The integrin binding site within FGF2 was identified as well; however the involvement of FGF2 in bridging FGFR1 and integrin αvβ3 has still to be determined [174].

Cell-cell contacts are complex signaling platforms that regulate behavior of neighboring cells and thus are strongly implicated in cancer. FGFRs are modulated by a number of different CAMs at the cell-cell interface. The FGFR-CAM interaction involves extracellular domains of these proteins, suggesting formation of complexes in cis and trans orientation. The FGFRs-CAMs interplay may adjust the strength of cell-cell attachment, which is relevant for migration of cancer cells and thus may constitute the target for future anticancer therapies. 

## 5. Novel Activities Acquired by FGFRs upon Binding to Specific Coreceptors 

Coreceptors are cell surface molecules that modulate the interaction of primary receptors with ligands. Usually, specific ligands require assembly of the ternary complexes involving ligand, receptor and coreceptor to initiate signal propagation. The perfect examples of FGFRs coreceptors are Klotho proteins that are necessary for endocrine FGFs (FGF19, FGF21, and FGF23) to trigger signaling. Functional FGFR signaling modules involve also specific polysaccharides, heparan sulfate (HS) chains, which stabilize receptor-ligand complexes. In this chapter we focus on coreceptors of FGFRs and their role in modulating FGFRs specificity and activity.

### 5.1. Heparan Sulfate Proteoglycans

**Figure 3:**
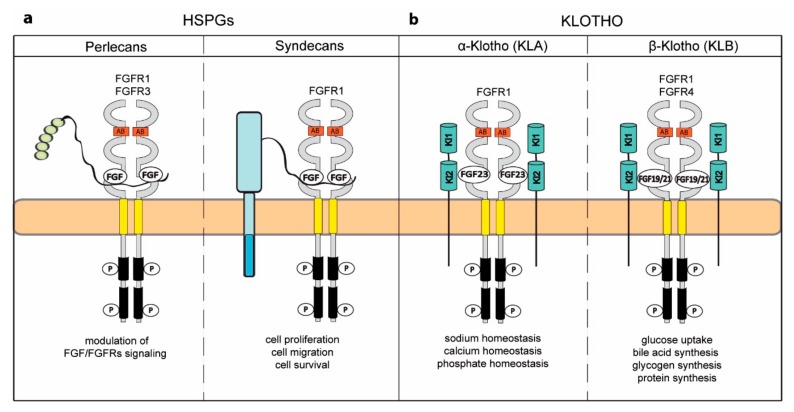
Involvement of coreceptors in the FGFRs signaling. (**a**) Heparan sulfate proteoglycans (HSPGs) provide polysaccharide chains that stabilize FGF-FGFR complexes and regulate availability of ligands. HSPGs are either integral membrane proteins (syndecans) or secreted glycoproteins (perlecans), which form ternary complexes with FGF-FGFR. (**b**) Klotho proteins α (KLA) and β (KLB) are necessary for FGF23 and FGF19/FGF21 signaling, respectively.

The formation of FGF-FGFR complexes requires presence of HS [175,176]. HS directly binds FGFs and FGFRs stabilizing the ternary complex and facilitating FGFR autophosphorylation [177]. HS chains are covalently attached to the serine residues of a subset of cell surface proteins, forming heparan sulfate proteoglycans (HSPGs). HSPGs are secreted into the extracellular space or are attached to the plasma membrane either via GPI anchor or transmembrane helix [178]. HSPGs participate in FGF signaling by regulating availability of FGFs to FGFRs and by adjusting the FGF-FGFR complex dynamics (Figure 3a) [179].

Perlecan is high molecular weight, multidomain HSPG ubiquitous in the extracellular space. The HS chains are attached to the N-terminal domain of perlecan [180]. Perlecan interacts with several FGFs, providing their storage in the extracellular matrix, thus adjusting their accessibility to FGFRs [181,182,183,184]. In the absence of FGF perlecan is able to bind FGFR3, but perlecan-FGFR1 interaction requires presence of the growth factor [182]. The ternary complexes involving FGFs (FGF20 or FGF18), perlecan, and FGFRs affect FGFRs signaling and resulting cellular response (Figure 3a) [181,185]. Interestingly, perlecan isolated from diverse tissues differentially modulates FGF/FGFRs signaling, highlighting the importance of HS structure for FGFRs [183]. 

Syndecans are composed of an N-terminal extracellular domain with attached several sugar chains, including HS, a single transmembrane helix and a C-terminal cytosolic tail [186]. The N-terminal domain of syndecans interacts with several proteins, including growth factors, extracellular matrix proteins and chemokines, the transmembrane helix facilitates oligomerization of syndecans, while the intracellular region interacts with numerous signaling and cytoskeletal proteins [187,188]. Syndecans via HS chains interact with FGFs and FGFRs with relatively low affinity, but still facilitating formation of ternary signaling complexes [189,190,191,192]. Syndecan-dependent modulation of FGF/FGFR complexes is relevant for cell proliferation, migration and survival (Figure 3a) [193,194,195,196]. The cellular trafficking of FGFRs is tightly regulated and constitutes a mechanism for adjustment of signaling pathways and cellular fate [29]. In endothelial cells syndecan-4 initiates the internalization of syndecan-4/FGF2/FGFR1 complexes via micropinocytosis that is independent of clathrin and dynamin, and involves RhoG and Rab4. The altered trafficking of FGFR1 changes kinetics of MAPK signaling important for survival of endothelial cells [197]. 

Another group of HSPGs that adjust cellular signaling pathways triggered by growth factors are GPI-anchored glypicans [198]. Glypican-1 interacts with FGFs, modulating their activity and accessibility for FGFRs [199,200,201]. However, in brain endothelial cells and in glioma cells the overexpression of glypican-1 facilitates mitogenic response triggered by FGF2 [202,203]. 

### 5.2. Klotho Coreceptors

The FGF family includes a subgroup of endocrine FGFs—FGF19, FGF21, and FGF23—which largely differ from canonical FGFs in their structure and mode of action. Endocrine FGFs circulate throughout the human body regulating numerous metabolic processes [204,205]. In contrast to canonical FGFs, endocrine FGFs display low affinity to FGFRs and cell surface heparans [206,207,208]. To form functional signaling complexes with FGFRs endocrine FGFs require obligatory coreceptors from Klotho family: α-Klotho (KLA) and β-Klotho (KLB) [209,210,211,212,213]. Klotho proteins are plasma membrane proteins containing two tandem KL1 and KL2 repeats with similarity to family 1 glucosidases in their extracellular region, a single transmembrane helix, and a short cytoplasmic tail [214,215]. 

KLA was discovered as a protein involved in aging process and is necessary for FGF23 signaling [211,214]. The obligate involvement of KLA in the formation of productive FGF23-FGFR1 signaling complex was enlightened by recent structural studies [216]. KLA interacts directly with FGFR1 and forms a high-affinity binding site for FGF23. FGF23 binds FGFR1 with its N-terminus, while the C-terminal region of FGF23 directly interacts with KLA, forming the KLA-FGF23-FGFR1 signaling complex (Figure 3b) [216]. Interestingly, dimerization of such complexes and receptor activation remain dependent on the binding of heparan sulfate [216]. This ternary complex acts mainly in kidneys, regulating sodium, calcium and phosphate homeostasis and its imbalance leads to various metabolic diseases, like acute and chronic uremia and premature aging [217,218,219,220,221,222,223,224,225].

KLB is a homologue of KLA that facilitates formation of signaling complexes containing FGFR--FGF19, mainly in hepatocytes, and FGFRs-FGF21 in adipocytes (Figure 3b) [226,227,228,229]. The molecular bases of FGFR-FGF19/FGF21-KLB signaling complex assembly largely resemble FGFR1-FGF23-KLA. KLB utilizes both KL1 and KL2 of the extracellular domain for direct binding to FGF19/FGF21 C-terminal domains [230,231]. The KLB-FGF19 complex binds FGFR1 and FGFR4, while KLB-FGF21 can form the ternary complex only with FGFR1 [227]. The dimerized KLB-FGF21-FGFR1 complexes in adipocytes induce catabolic processes, stimulate glucose uptake, and improve insulin sensitivity [232]. Noteworthy, acting as a fasting hormone, FGF21 significantly extends lifespan [233,234]. In hepatocytes the KLB-FGF19-FGFR4 complexes are formed in response to feeding and downregulate synthesis of bile acid [235,236]. Additionally, these complexes contribute to the regulation of blood glucose level by stimulating synthesis of glycogen [237,238]. The dysregulation of FGF19/FGF21 is implicated in metabolic diseases, aging, and cancer [217,239,240,241]. 

## 6. Modulation of FGFRs by Other Cell Surface Proteins

There are plasma membrane proteins that interact with FGFRs but cannot be assigned to the above described categories. One of them is transforming growth factor β receptor III (TGFBRIII), which is also known as betaglycan. It is a coreceptor of TGFBRI and TBFBRII that lacks an intracellular kinase activity [242]. The interaction between TGFBRIII and FGFR1 was demonstrated by coimmunoprecipitation in neuroblastoma. The TGFBRIII-FGFR1 interaction is stimulated by FGF2 and the assembly of ternary complexes enhances FGF2 signaling and promotes neuronal differentiation [243]. In addition, FGF2 binds to the glysocaminoglycan chains (GAG) present on the extracellular region of TGFBRIII, which may regulate availability of the FGF2 to FGFRs on the cell surface [244].

Another FGFRs’ interactor is Sef (similar expression to fgf genes), a receptor-like protein composed of an extracellular region containing the FN3 domain, a single transmembrane helix and an intracellular domain with similarity to the interleukin 17 receptor [245]. Besides membrane bound Sef, secreted and cytosolic isoforms of Sef are generated [246]. The expression of Sef is induced by FGF signaling in various cell types [245,247,248,249]. The interaction of various Sef isoforms with intracellular region of FGFRs was demonstrated with coimmunoprecipitation [246,249,250,251,252,253]. Sef is an inhibitor of FGFR-dependent signaling acting either directly at the level of the receptor and/or on downstream intracellular kinases [254]. FGFR-dependent activation of ERK/MAPK and Akt is blocked by Sef, resulting in inhibition of cell proliferation [250,255]. Sef can also induce apoptosis and affect FGF-induced differentiation in various cell types [255]. Notably, the FGFRs-Sef interplay was implicated in prostate cancer [256,257].

## 7. Conclusions

The cellular fate is very rarely determined by isolated signaling units. Instead, it is rather a result of extensive cross-communication between numerous diverse ligand/receptor systems. Secreted FGFs and their receptors are well studied signaling molecules. However, a number of recent reports largely changed the view about FGFs/FGFRs as separate signaling modules. FGFs/FGFRs are integrated into the complex cellular signaling at many levels and are subjected to diverse regulatory mechanisms. The cross-talk between FGFRs and other cell surface receptors, adhesion molecules, and coreceptors effectively modulates cellular processes such as proliferation, motility, differentiation, and death. The list of FGFRs binding partners within the plasma membrane is expanding; however it is still far from complete. As FGFRs expose large domains towards the extracellular space and the cytosol, the activity of these receptors might be further modulated by currently unknown secreted and/or intracellular proteins, respectively. Certainly, further studies aiming on the identification of novel FGFRs binding proteins and deciphering the relevance of FGFRs’ complexes are required. Moreover, the application of complementary in vitro and in vivo experimental approaches is required for the validation and in-depth characterization of identified interactions. Structural data revealed the molecular mechanism of FGFR tyrosine kinase activation facilitating the design of diverse FGFR small molecule inhibitors that are currently tested as anticancer drugs [258]. Similarly, understanding how FGFRs cooperate with other cell surface receptors may lead to the development of novel inhibitors targeting FGFR-dependent processes. 

As FGFRs are embedded in the plasma membrane, the activity and distribution of these receptors can be additionally affected by properties of the cell membrane (membrane composition, organization, curvature, etc.). Additionally, the alternative splicing of FGFRs and partner proteins may constitute another regulatory mechanism of the assembly of multiprotein signaling complexes. Further studies in this direction are unquestionably required. The spatiotemporal regulation of FGFRs constitutes another way to adjust cellular signaling. Some binding partners affect cellular trafficking of FGFRs, influencing selected transport mechanism and subcellular destination of the receptors. This in turn affects the kinetics and specificity of signaling and modulates cellular response. As FGFRs and number of partner proteins are implicated in various diseases including cancer, the deeper understanding of the interplay between FGFRs and other components of the cell membrane may facilitate treatment of life-threatening diseases.

## Abbreviations

5-HT1A	5-hydroksytriptamine receptor 1A
8-OH-DPAT	7(Dipropylamino)-5,6,7,8-tetrahydronaphthalen-1-ol
A2AR	adenosine receptor
AJ	adherens junction
AKT	protein kinase B
ATP	adenosine triphosphate
BRET	bioluminescence resonance energy transfer
CAMs	cell adhesion molecules
CB1R	cannabinoid receptor 1
CNS	central nervous system
Dlg-1	disks large homolog 1
EGFs	epidermal growth factors
EGFRs	epidermal growth factor receptors
EMT	epithelial to mesenchymal transition
Eph	ephrin
ERK1/2	extracellular regulated kinases 1/2
FGFs	fibroblast growth factors
FGFRs	fibroblast growth factor receptors
FGFRL1	fibroblast growth factor receptor like 1
FLRTs	fibronectin leucine-rich transmembranes
FN3	fibronectin type III
FRET	Förster Resonance Energy Transfer
FRS2	fibroblast growth factor receptor substrate 2
GAG	glysocaminoglycan
GPCRs	G-protein-coupled receptors
GPI	glycosylphosphatidylinositol
HS	heparan sulfate
HSPGs	heparan sulfate proteoglycans
IGFR	insulin-like growth factor receptor
JM	juxtamembrane
KLA	α-klotho
KLB	β-klotho
L1-CAM	L1 cell adhesion molecule
LNS	laminin, neurexin, sex hormone binding globulin
LPR	leucine-rich repeat domain
mAChR	muscarinic acetylcholine receptor
MAPK	mitogen-activated protein kinase
MOR	mu-opioid receptor
mTOR	mammalian target of rapamycin
N-CAMs	neural cell adhesion molecules
NEGR1	neuronal growth regulator 1
NFs	neurofascins
Np55	neuroplastin 55
OPCML	opioid binding protein cell adhesion molecule
PDGFs	platelet-derived growth factors
PDGFRs	platelet-derived growth factor receptors
PI13K	phosphoinositide 3-kinase
PKC	protein kinase C
PLA	proximity ligation assay
PLCɣ	phospholipase Cɣ
RTKs	receptor tyrosine kinases
SAM	sterile alpha motif
Sef	similar expression to fgf genes
SPA	solid-phase assay
SPR	surface plasmon resonance
STAT	signal transducer and activator of transcription
TGFs	transforming growth factors
TGFBRs	transforming growth factor receptors
Y2H	yeast two-hybrid
